# Interactive Effects of Elevated CO_2_ Concentration and Irrigation on Photosynthetic Parameters and Yield of Maize in Northeast China

**DOI:** 10.1371/journal.pone.0098318

**Published:** 2014-05-21

**Authors:** Fanchao Meng, Jiahua Zhang, Fengmei Yao, Cui Hao

**Affiliations:** 1 Institute of Eco-Environment and Agro-Meteorology, Chinese Academy of Meteorological Sciences, Beijing, China; 2 College of Atmospheric Science, Nanjing University of Information Science & Technology, Nanjing, China; 3 Key Laboratory of Digital Earth Science, Institute of Remote Sensing and Digital Earth, Chinese Academy of Sciences, Beijing, China; 4 Key Laboratory of Computational Geodynamics, Chinese Academy of Sciences, Beijing, China; University of Antwerp, Belgium

## Abstract

Maize is one of the major cultivated crops of China, having a central role in ensuring the food security of the country. There has been a significant increase in studies of maize under interactive effects of elevated CO_2_ concentration ([CO_2_]) and other factors, yet the interactive effects of elevated [CO_2_] and increasing precipitation on maize has remained unclear. In this study, a manipulative experiment in Jinzhou, Liaoning province, Northeast China was performed so as to obtain reliable results concerning the later effects. The Open Top Chambers (OTCs) experiment was designed to control contrasting [CO_2_] i.e., 390, 450 and 550 µmol·mol^−1^, and the experiment with 15% increasing precipitation levels was also set based on the average monthly precipitation of 5–9 month from 1981 to 2010 and controlled by irrigation. Thus, six treatments, i.e. C_550_W_+15%_, C_550_W_0_, C_450_W_+15%_, C_450_W_0_, C_390_W_+15%_ and C_390_W_0_ were included in this study. The results showed that the irrigation under elevated [CO_2_] levels increased the leaf net photosynthetic rate (*P*_n_) and intercellular CO_2_ concentration (*C*_i_) of maize. Similarly, the stomatal conductance (*G*_s_) and transpiration rate (*T*_r_) decreased with elevated [CO_2_], but irrigation have a positive effect on increased of them at each [CO_2_] level, resulting in the water use efficiency (*WUE*) higher in natural precipitation treatment than irrigation treatment at elevated [CO_2_] levels. Irradiance-response parameters, e.g., maximum net photosynthetic rate (*P*_nmax_) and light saturation points (*LSP*) were increased under elevated [CO_2_] and irrigation, and dark respiration (*R*_d_) was increased as well. The growth characteristics, e.g., plant height, leaf area and aboveground biomass were enhanced, resulting in an improved of yield and ear characteristics except axle diameter. The study concluded by reporting that, future elevated [CO_2_] may favor to maize when coupled with increasing amount of precipitation in Northeast China.

## Introduction

The CO_2_ concentration ([CO_2_]) in the atmosphere is about 390 µmol·mol^−1^ as a consequence of fossil fuel combustion and deforestation, which is predicted to reach 550 µmol·mol^−1^ by the middle of this century [1]. Elevated [CO_2_] is an important abiotic factor, and has significant fertilization effects on crops. Extensive previous studies have reported that elevated [CO_2_] significantly improved water use efficiency, lower transpiration rate, shorten maize growth period, and increased plant height, leaf number, leaf area, growth rate and yield [2]–[12]. In addition, the increasing of atmospheric [CO_2_] affects precipitation balance, which can change the seasonal precipitation distribution [13]. It has been estimated that this effect would bring about a 10% increase or decrease in water resources at different areas [14]. The global annual average precipitation increase is about 2% since the beginning of the 20^th^ century [15]–[16], and this rise over the area of 30°–85°N has shown a 7%–12% increase [17]. It has been predicted that the rainfall decrease will be noticed in middle-and-lower regions of Yangtze River (24°N–34°N,108°E–122°E), while the rain belts are likely to move towards north of China and precipitation would increase in Northeast China in the future [18]. The crop growth of Northeast China will likely be affected by both elevated [CO_2_] and increasing precipitation, which are important abiotic factors that directly or indirectly affect crop growth, physiological processes and productivity. Thus, it is necessary to understand the interactive effects of elevated [CO_2_] and increasing precipitation on crop growth in Northeast China under future climate change.

In fact, lots of studies have been focused on the interactive effects of elevated [CO_2_] and other environmental factors on plant growth. The study of the interactive effects of elevated [CO_2_] and temperature indicated that the effects on photosynthesis and growth in C_4_ species are obvious [19]–[22]. FACE (Free Air Carbon-dioxide Enrichment) and chamber experiment have demonstrated that the interactive effects of elevated [CO_2_] and drought stress have an increase in the leaf water-use efficiency [23]–[24], and more recent evidence shows that maize will benefit from the increase in [CO_2_] under drought condition [25]–[29]. Also, the studies of the interactive effects of elevated [CO_2_] and light on plant found that high light have a great effect on net photosynthesis in condition of elevated [CO_2_] [30]–[31]. Regarding the interactive effects of elevated [CO_2_] and Ozone (O_3_), studies showing that elevated [CO_2_] inhibits adverse effects of O_3_ and increased trees seedling stem diameters at low O_3_ [32]–[35]. Moreover, the interactive effects of elevated [CO_2_] and soil nutrition have been investigated. For example, the studies of the interactive effects of elevated [CO_2_] and nitrogen (N) indicated that there is a positive CO_2_×N interaction for grain yield of rice [36]–[38], while the research on the interactive effects of elevated [CO_2_] and potassium (K) found that plants grown under elevated [CO_2_] are more sensitive to K deficiency with higher leaf critical K levels [39]. Further, there are lots of studies involving the interactive effects of elevated [CO_2_] and other factors (e.g., Nacl-salinity, plant diversity) have been reported [40]–[41]. However, the interactive effects of elevated [CO_2_] and increasing precipitation on photosynthesis and yield of maize are not well understood. In particular, there has been no detailed study evaluating the interactive effects of elevated [CO_2_] and increasing precipitation on photosynthesis efficiency, water use efficiency and yield of maize in Northeast China.

Northeast China (38°N–56°N,120°E–135°E) is located in the middle-high latitudes and east of the Eurasian continent, which has a cultivated land area of 21.53 million hm^2^, accounted for 16.6% of the country's total cultivated areas [42]. The summer is warm and short, and the annual precipitation is 400–800 mm. The precipitation from July to September is accounting for 60% of the annual precipitation. Moreover, Northeast China has fertile black soil, belonging to one of the three pieces of black soil in the world [43], hence, which is the biggest commercial grain production base and provides 30–35 million tons of commercial grain to country every year [44]. Therefore, it plays an important role to stabilize the grain market and keep sustainable development of China's national economy. In Northeast China, maize (*Zea mays L.*) is the major cultivated crop, and its yield has accounted for about 1/3 of the national total maize yield [45]. The growth of maize requires more water, which yields tend to decrease if water deficit occurred during the key growing stages (e.g., silking stage) [46]. The precipitation in Northeast China can meet the water requirements of maize in most of the years, but slight drought has been discovered to occur in some of the past years. Therefore, for the rain-fed maize in Northeast China, precipitation is a very important climatic factor. If the water deficit occurred at silking stage of maize will cause disaccord flowering season, and then affect the pollination and seed formation, resulting in maize yield reduction.

To examine the interactive effects of elevated [CO_2_] and increasing precipitation on maize in Northeast China, we conducted an Open Top Chambers (OTCs) experiment under the combined effects of elevated [CO_2_] and precipitation in Jinzhou, Liaoning province during maize growing season (May to September) in 2013. Firstly, we tested the response of leaf gas exchange parameters (e.g., *P*_n_, *T*_r_) and irradiance-response parameters (e.g., *P*_nmax_, *LSP*) to the combined elevated [CO_2_] and increasing precipitation. Secondly, we examined the change in the growth parameters of maize (e.g., leaf area, aboveground biomass), yield and ear characters (e.g., ear length, ear diameter). The results of this study would be crucial for evaluating the possible consequences of climate change on crop photosynthetic capacity and yield in Northeast China, and may help inform regulatory policies to cope with the future climate change.

## Materials and Methods

### Experimental site

This study site is located at Jinzhou Ecological and Agricultural Meteorological Experiment Center (41°09′N,121°10′E,27.4 m a.s.l.) in Liaoning province of China, which is a warm temperature monsoon climate zone. The mean annual precipitation is 568.8 mm, and the mean annual temperature is 9.1°C. The annual frost-free period is approximately 180 d in duration, with an annual accumulated activity temperature is 3700°C·d. The site has typical brown soil, and the soil pH is approximately 6.3. The soil organic matter and total N are 6.41–9.43 g/kg and 0.69 g/kg, respectively [47].

### Open Top Chamber design

**Figure 1:**
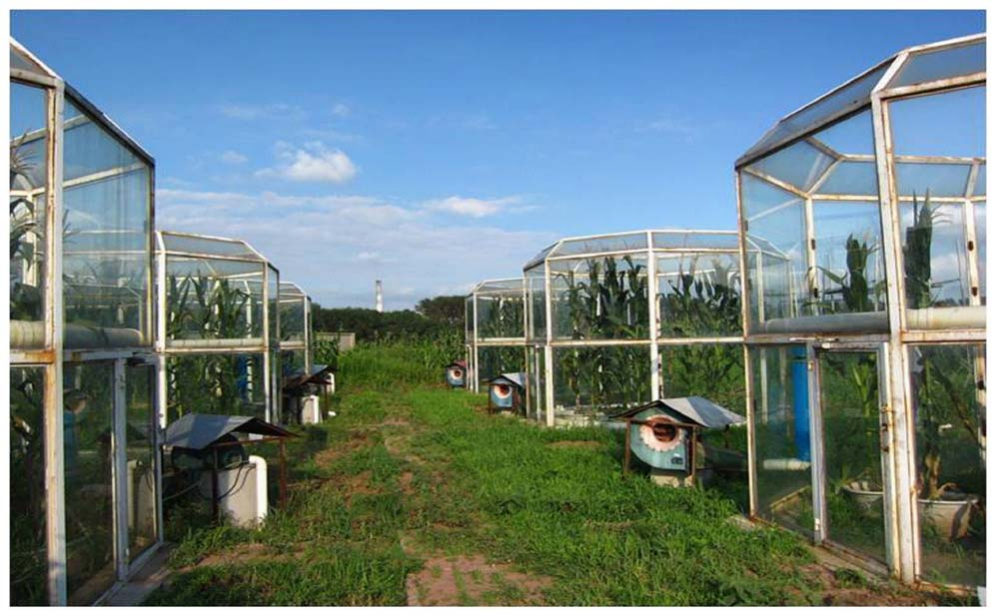
View of Open Top Chambers on 21 July 2013 at Liaoning province, China.

Three pairs of Open Top Chambers (OTCs), each 3.5 m high with an octagonal ground surface area of 11.73 m^2^ were constructed. An inclined plane of 45° (inward on upper side of chambers) was provided for reducing gas escape from the top. The set up was completed in May 2011 (Fig. 1).

Additionally, the OTCs were constructed with a 5.5-m-wide buffer zone between them to prevent mutual shading. Carbon dioxide was supplied to the chambers through a pipe with pinholes connected to industrial carbon dioxide cylinders (liquid carbon dioxide, purity was 99.99%, supplied by Anjin Gas Corporation) outside the chambers. There was an exchange fan of each chamber, which mixed the entered carbon dioxide and fresh air from outside, then transported by pipe and well distributed in the entire chamber by the octagonal-pipe with holes, and the gas would discharge from the opening on top and put in air circulation. Carbon dioxide was supplied for 24 hours a day and the [CO_2_] was monitored by taking constant measurements with an infrared gas analyzer.

### Experimental design

**Figure 2:**
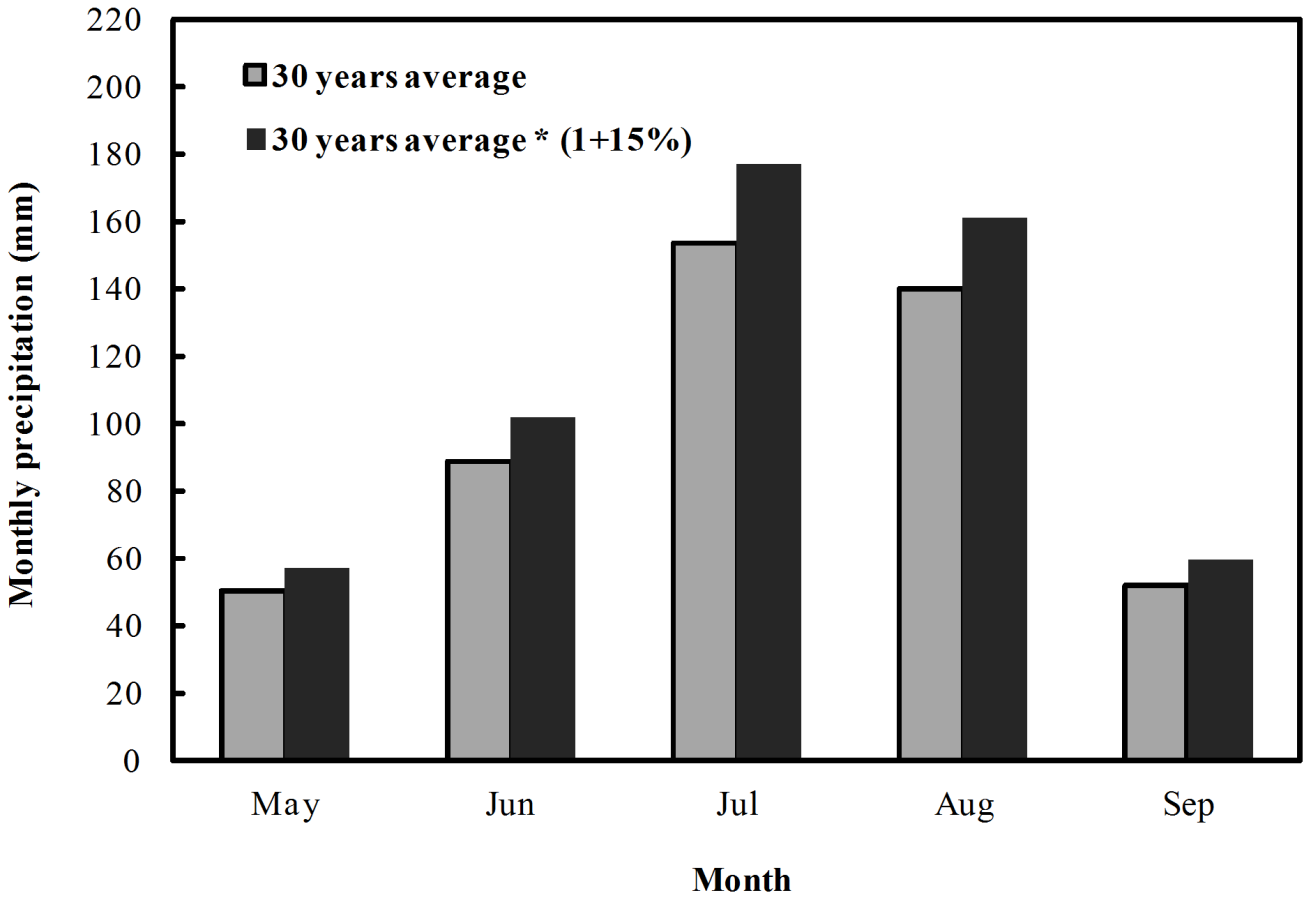
Monthly average precipitation during 1981–2010 years in the growing season of Maize in study area. Gray bars indicate the regional monthly averages precipitation from 1981 to 2010, and black bars indicate increased 15% precipitation based on gray bars.

The effects of elevated [CO_2_] and precipitation on photosynthesis, growth, yield and ear characteristics of maize were examined in the chamber experiments. Considering the present ambient [CO_2_] and projected increasing [CO_2_] levels in next several decades from IPCC (2007) [1], three [CO_2_] levels were conducted with the OTC experiments including 390 µmol·mol^−1^ (C_390_), 450 µmol·mol^−1^ (C_450_) and 550 µmol·mol^−1^ (C_550_). According to 5–9 month average monthly precipitation in Jinzhou during 1981–2010 (see Fig. 2) and the prediction of increasing rainfall in Northeast China [18], [48]–[49], two levels of watering including natural precipitation (W_0_) and precipitation increased by 15% (W_+15_) were designed. With this, maize, grown from seeds, was subjected to 6 different combined treatments (Table 1).

**Table 1:** Treatments performed in OTCs.

Treatments	Description
C_550_W_+15%_	Elevated [CO_2_] concentration (550 µmol·mol^−1^) and Increased 15% of precipitation
C_550_W_0_(CK)	Elevated [CO_2_] concentration (550 µmol·mol^−1^) and Natural precipitation
C_450_W_+15%_	Elevated [CO_2_] concentration (450 µmol·mol^−1^) and Increased 15% of precipitation
C_450_W_0_(CK)	Elevated [CO_2_] concentration (450 µmol·mol^−1^) and Natural precipitation
C_390_W_+15%_	Ambient [CO_2_] concentration (390 µmol·mol^−1^) and Increased 15% of precipitation
C_390_W_0_(CK)	Ambient [CO_2_] concentration (390 µmol·mol^−1^) and Natural precipitation

Three pairs of OTCs were used, and within each pair one was randomly assigned to receive the control (390 µmol·mol^−1^) and the others as the elevated [CO_2_] (450 and 550 µmol·mol^−1^). Every chamber was designed with two watering gradient (0 and +15%), and each gradients with 7 pots (50.5 cm (diameter)×32.5 cm (height)), producing 14 plots per treatment with a total of 84 plots. Based on the local long-term (1981–2010) monthly mean precipitation, watering volumes of each pot were 20.43 L, 35.43 L, 32.21 L and 11.96 L in the W_+15%_ treatment plots from June to September, respectively. It was divided into everyday average irrigation, directed into the pots in the morning and evening daily, and covering the raining days too.

The maize cultivar used in this study was Danyu 39, and now it is widely planted in Northeast China. Three maize seeds were planted in the pots using field soil on 10 May 2013, with final seeding at four-leaf stage. On 1 June, the 84 pots containing plants were moved into the chambers randomly, and each chamber contained 14 pots. Meanwhile, the levels of [CO_2_] and water supplied were under monitored and controlled until harvest time (15 September).

### Rainfall date and relative soil moisture measurements

The rainfall data were obtained from the Jinzhou weather station. The relative soil moisture (RSM) was calculated by equation (1) given below [50]: 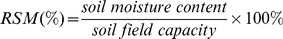
(1)


The soil moisture content was measured at a 0–20 cm soil depth. The soil samples of each treatment was collected and recorded as fresh weights and the samples were dried in an oven at 105°C for at least 48 hours. The soil moisture content was then measured using equation (2) given below: 
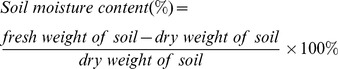
(2)


And the soil field capacity in this study was used the value of 21%, which according to the average value of soil field capacity in the last few years at this study site [51].

### Gas exchange measurements of maize

Measurements were made between 8:30 a.m. and 11:30 a.m. (local time) from 24–26 July, 2013 (at the maize silking stage). Three representative plants were chosen for per treatment and the middle of the ear-leaves was measured, then the averages were taken. Gas exchange measurements were conducted using portable gas exchange systems (LI-6400, LI-Cor, Lincoln, NE, USA). The [CO_2_] in the leaf chamber was controlled by the LI-Cor CO_2_ injection system, and the built-in LED lamp (red/blue) supplied the irradiance. Temperature in leaf chamber was set at 30°C, and the actual temperature of the leaf chamber ranged from 29 to 33°C. The vapour pressure deficit on the leaf surface (VpdL) was between 2.9 and 3.4 kPa, and the flow control was at 500 µmol·s^−1^. The lamp settings across the series of 2000, 1600, 1200, 1000, 800, 600, 400, 200, 150, 100, 80, 50, 20 and 0 µmol·m^−2^·s^−1^, and the measurements were recorded after equilibrium was reached. Each individual curve took approximately 30 min to complete.

*P*_n_ curve fitting and analyzed of parameters were performed using the modified rectangular hyperbolic model by Ye and Yu [52]–[53], and its expression and correlative equations is as given below: 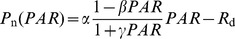
(3)where *PAR* is irradiance, *α* is the initial slope of irradiance-response curve of photosynthesis when irradiance approaches to zero, *β* and *γ* are coefficients which are independent of *PAR*, *R*_d_ is dark respiration (µmol·m^−2^·s^−1^). From these parameters, we can calculate *P*_nmax_ (maximum net photosynthetic rate, µmol·m^−2^·s^−1^), *LSP* (light saturation point, µmol·m^−2^·s^−1^), *LCP* (light compensation point, µmol·m^−2^·s^−1^) and *φ*_c_ (quantum efficiency of the light compensation point, mol·mol^−1^) using equations (4)–(7) as described by Han et al. [54]. 
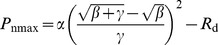
(4)

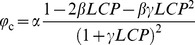
(5)

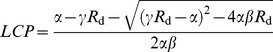
(6)

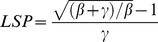
(7)


*T*_r_ (transpiration rate, mol·m^−2^·s^−1^), *G*_s_ (stomatal conductance, µmol·m^−2^·s^−1^) and *C*_i_ (intercellular CO_2_ concentration, µmol··mol^−1^) were also measured at the same irradiance, temperature and vapour pressure when the measurements of *P*_n_ were conducted. Additionally, *WUE* (water use efficiency, µmol·mol^−1^) was calculated as *P*_n_/*T*_r_.

### Growth and harvesting of maize

Maize growth stages were recorded throughout the growing season. The plant height, ear height, stem diameter, leaf area and aboveground biomass were measured at the silking stage of the maize. Leaf area of each plant was determined with long-width coefficient method (length × width × 0.75). Aboveground biomass was obtained by dry weight. Before weighing, three plants from each treatment were separated into stem, leaf and grain. This was as a result of the need to shrivel it in oven at 105°C for 45 min and drying to constant weight at 85°C for at least 48 h.

At maturity stage, ten plants of each treatment were harvested for the yield components. The grains from each ear of maize were threshed by hand after air dried and weighed. The measured ear characteristics include: ear length, ear diameter, ear weight, 100-kernel weight, rows per ear, kernel number, shriveled kernels, bare-tip length and axle diameter. The yield of each plant was calculated by 14% moisture content of grain.

During sampling, three representative plants of each treatment were randomly selected for measurement.

### Statistical analysis

In this study, statistical significance of growth and yield components was tested at 0.05 probability level (*P*<0.05) following the DUNCAN test, performed using DPS 7.05 (Data-processing System, Zhejiang University, China). Irradiance-response curve fitting and parameter analysis based on modified rectangular hyperbolic model. The two-way analysis of variances (ANOVAS) were used to examine the interactive effects of elevated [CO_2_] and irrigation on growth parameters and yield of maize, and statistical significance were set at *P*<0.05 and *P*<0.01. These statistical analyses were conducted with SPSS 17.0 software (SPSS Institute Incorporated, Chicago, Illinois, USA). The standard deviation (S.D.) was calculated to compare the treatment means.

## Results

### Rainfall data and relative soil moisture

The variations of 5–9 month average monthly precipitation in Jinzhou during 1981–2010 are as shown in this work (Fig. 2). The work indicated that the amount of rainfall was maximum in July (176.93 mm) and less in May (57.43 mm).

**Figure 3:**
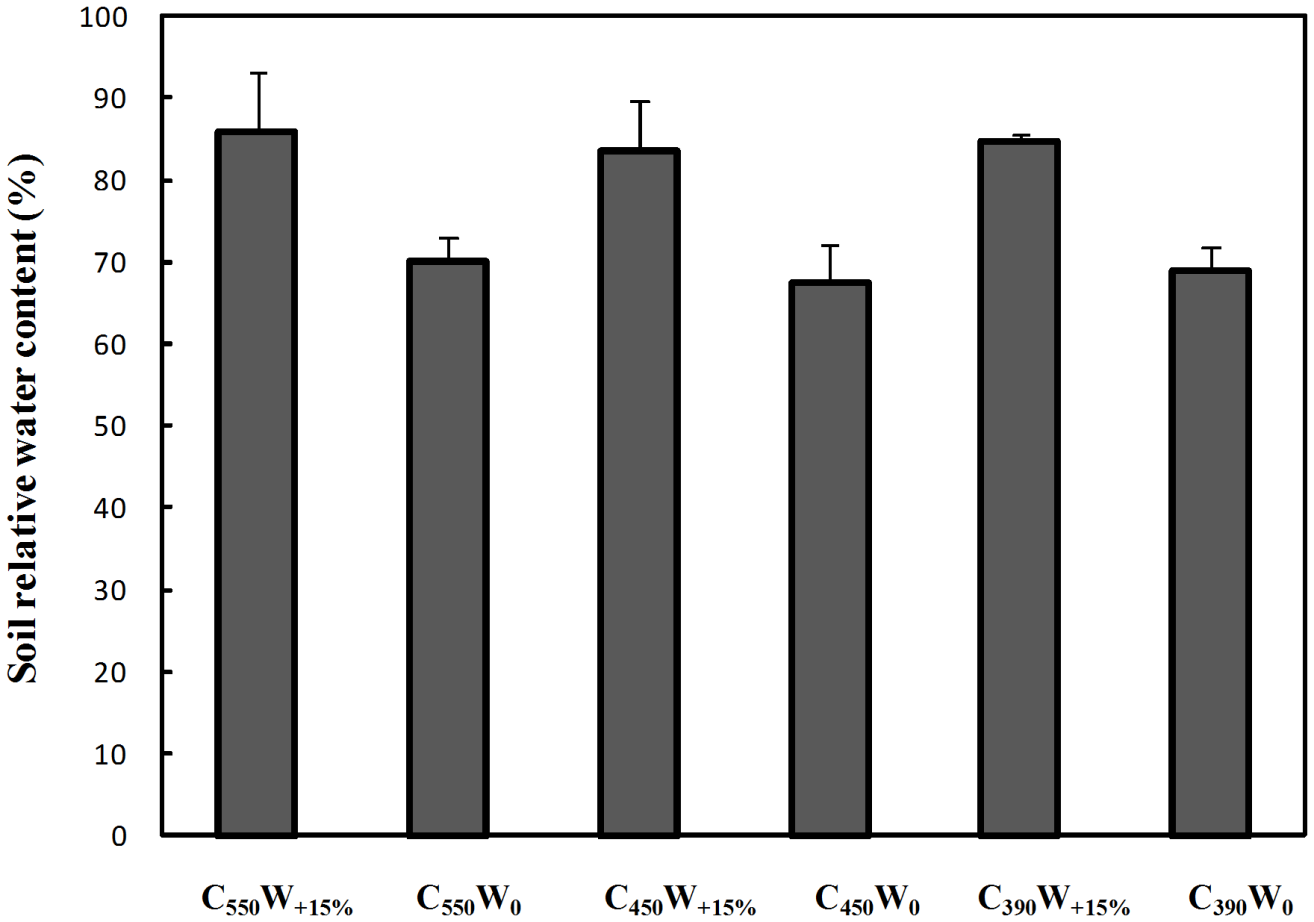
The relative soil moisture (means ± SD) (n = 3) in six treatments (C_550_W_+15%_, C_550_W_0_, C_450_W_+15%_, C_450_W_0_, C_390_W_+15%_ and C_390_W_0_) in silking stage of maize under effects of elevated [CO_2_] and irrigation.

The relative soil moisture of irrigation treatment was higher than that of the natural precipitation treatment at each [CO_2_] level (Fig. 3). The range of relative soil moisture in irrigation treatment was about 68.55%–69.84% and that of natural precipitation was about 56.68%–58.32%. According to the national standard of the Classification of Meteorological Drought (GB/T20481-2006) [50], we can see that the natural precipitation treatments are in-fact under slight drought.

### Photosynthetic gas exchange parameters of maize

**Figure 4:**
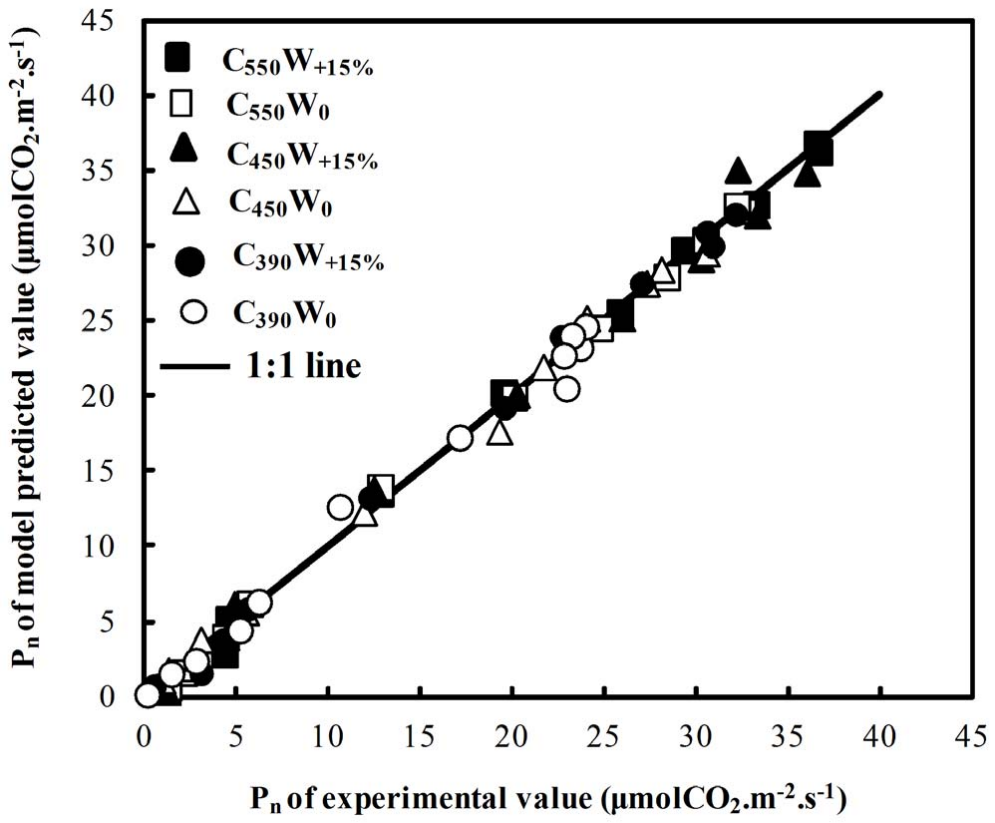
*P*_n_ of experimental value comparing with *P*_n_ of model predicted value. The 1∶1 line indicates *P*_n_ of experimental value equals to the predicted value.

**Figure 5:**
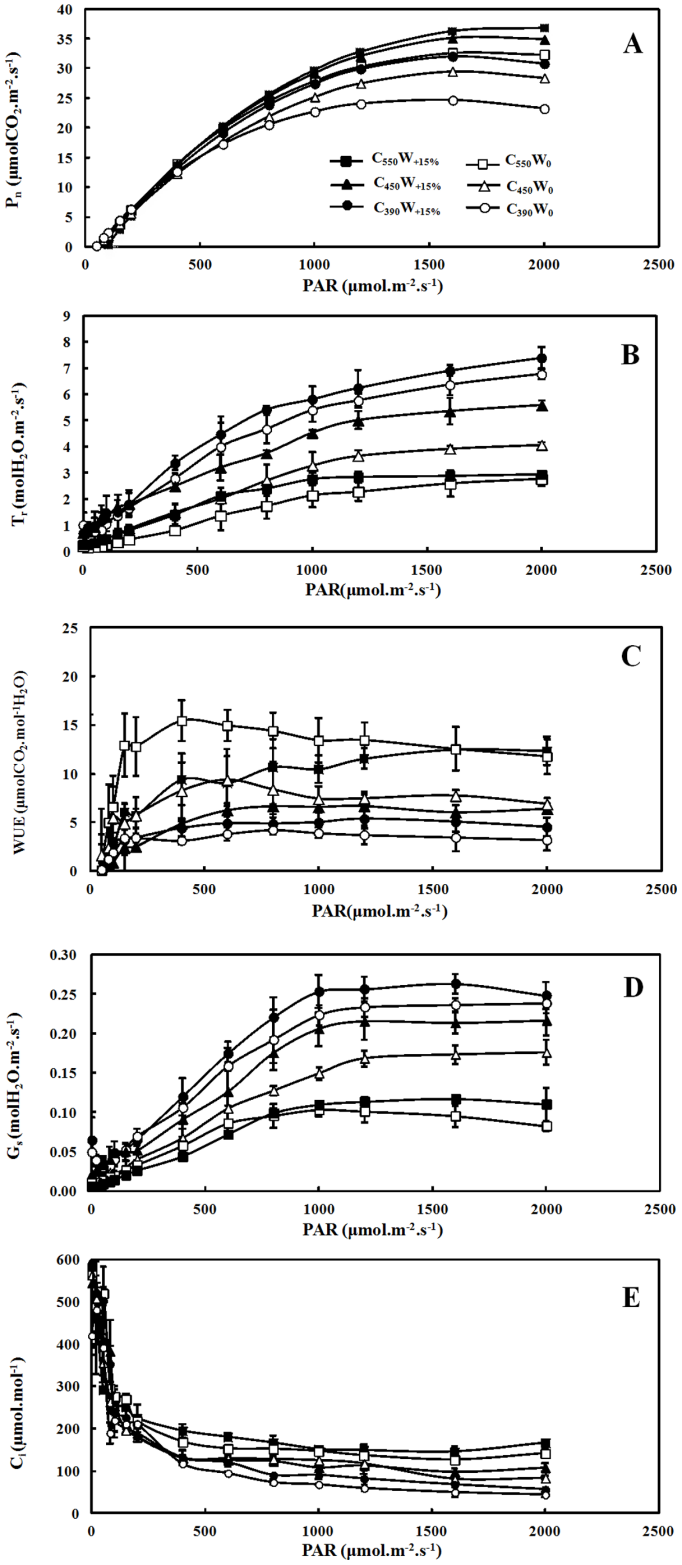
Dynamic curves of (A) net photosynthetic rate (*P*_n_), (B) transpiration rate (*T*_r_), (C) water use efficiency (*WUE*), (D) stomatal conductance (*G*_s_) and (E) intercellular CO_2_ concentration (*C*_i_) (means ± SD) (n = 3) in six treatments (C_550_W_+15%_, C_550_W_0_, C_450_W_+15%_, C_450_W_0_, C_390_W_+15%_ and C_390_W_0_) in silking stage of maize under effects of elevated [CO_2_] and irrigation.

According to previous studies, the relationship between photosynthetic rate and irradiance could be well described by modified rectangular hyperbolic model [52]. This model was used to obtain the irradiance-response curve (Fig. 5 A), with R^2^>0.99 for all treatments, meanwhile the characteristic parameters were calculated from table 2. From the comparison between *P*_n_ of experimental value and model predicted value (Fig. 4), it could be seen that there was a good agreement between them.

**Table 2:** Parameters of irradiance-response curves of maize under effects of elevated [CO_2_] and different irrigation.

Treatments	*P*_nmax_ /(µmol·m^−2^·s^−1^)	*LSP*/(µmol·m^−2^·s^−1^)	*LCP* /(µmol·m^−2^·s^−1^)	*φ*_c_ /(mol·mol^−1^)	*R*_d_ /(µmol·m^−2^·s^−1^)	R^2^
C_550_W_+15%_	36.8702	1884.3777	94.8387	0.0544	5.1268	0.997
C_550_W_0_(CK)	32.7945	1740.8274	68.3083	0.0530	3.5925	0.998
C_450_W_+15%_	35.3561	1768.8612	62.8339	0.0483	3.0519	0.994
C_450_W_0_(CK)	29.5133	1650.8724	54.2541	0.0417	2.2788	0.997
C_390_W_+15%_	32.0309	1643.9243	66.9802	0.0469	3.1668	0.993
C_390_W_0_(CK)	24.6966	1501.2016	46.8651	0.0482	2.2332	0.992

Abbreviations are: *P*_nmax_ - maximum net photosynthetic rate, *LSP* - light saturation point, *LCP* - light compensation point, *φ*_c_ - quantum efficiency of the light compensation point, *R*_d_ - dark respiration.

Figure 5 showed the dynamic changes of *P*_n_, *T*_r_, *WUE*, *G*_s_ and *C*_i_ with *PAR* increased during silking stage of maize under interactive effects of elevated [CO_2_] and irrigation. With increased of *PAR*, *P*_n_ curves of six treatments increased with elevated [CO_2_] and irrigation. Similarly, when *PAR* is above 1500 µmol·m^−2^·s^−1^, *P*_n_ curves closed to saturation and becomes stable. The order of six treatments were: C_550_W_+15%_>C_450_W_+15%_>C_550_W_0_>C_390_W_+15%_>C_450_W_0_>C_390_W_0_ (Fig. 5A); Whereas all the *T*_r_ curves decreased with elevated [CO_2_], and were much lower in natural precipitation than irrigation treatment at each [CO_2_] level (Fig. 5B). *WUE* showed higher values with elevated [CO_2_], and natural precipitation treatment showed higher than irrigation treatment at elevated [CO_2_] levels (Fig. 5C). However, *G*_s_ curves showed opposite trend. The *G*_s_ curves were lower with elevated [CO_2_] and rose with irrigation at the same [CO_2_] levels (Fig. 5D). There were high trends of *C*_i_ under elevated [CO_2_], and the irrigation treatments were higher with the increased amounts in *PAR* at each [CO_2_] level (Fig. 5E).

The *P*_n_ curves were fitted and the parameters were generated by the modified rectangular hyperbolic model. The parameter *P*_nmax_ of the ear-leaves for maize was increased by 12.43%, 19.80% and 29.70% under irrigation conditions above the natural precipitation at 390, 450 and 550 µmol·mol^−1^ [CO_2_] levels, respectively. *φ*_c_ was not affected by irrigation and elevated [CO_2_] (Table 2). Irrigation increased *LSP* by 8.25%, 7.60% and 9.97% at 390, 450 and 550 µmol·mol^−1^ [CO_2_] levels, respectively, but *LCP* increased by 38.73%, 15.81% and 42.92%, and *R*_d_ increased by 42.71%, 33.93% and 41.81% under the same conditions (Table 2).

### Growth and development of maize

**Figure 6:**
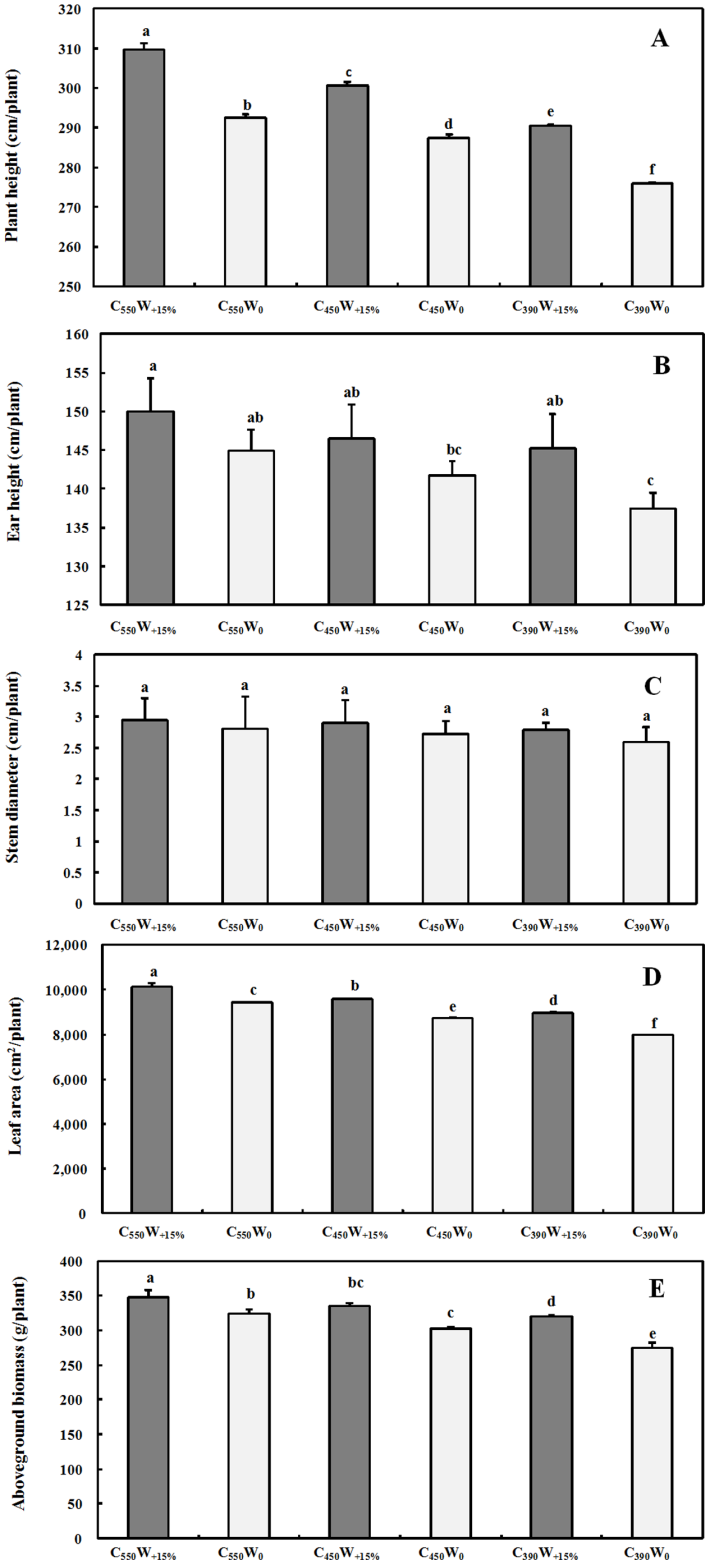
Changes of (A) plant height, (B) ear height, (C) stem diameter, (D) leaf area and (E) aboveground biomass (means ± SD) (n = 3) in six treatments (C_550_W_+15%_, C_550_W_0_, C_450_W_+15%_, C_450_W_0_, C_390_W_+15%_ and C_390_W_0_) in silking stage of maize under effects of elevated [CO_2_] and irrigation. Different lower cases letters indicated significant difference (P<0.05).

Plant height, ear height, stem diameter, leaf area and aboveground biomass of maize all had increasing trend under elevated [CO_2_] and irrigation, while C_390_W_0_ always at its lowest value (Fig. 6). plant height of C_550_W_+15%_, C_450_W_+15%_ and C_390_W_+15%_ were significantly higher than C_550_W_0_, C_450_W_0_ and C_390_W_0_ by 5.28%, 4.60% and 5.86%, respectively (*P*<0.05; Fig. 6A), while ear height were higher by 5.69%, 3.34% and 3.50%, respectively, and the same trend as plant height (*P*<0.05; Fig. 6B). There were no significant differences of stem diameter for all treatments at silking stage, but C_390_W_0_ was observed to be low (*P*<0.05; Fig. 6C). There was a significant interactive effects for elevated [CO_2_] and irrigation in plant height (*P*<0.05; Table 4), but no significant interactive effects in ear height and stem diameter (*P*<0.05; Table 4). Irrigation significantly increased the leaf area by 12.04%, 9.90% and 7.75% at 390, 450 and 550 µmol·mol^−1^ [CO_2_] levels, respectively (*P*<0.05; Fig. 6D). In addition, irrigation significantly increased aboveground biomass by 16.66%, 10.75% and 7.65% at 390, 450 and 550 µmol·mol^−1^ [CO_2_] levels, respectively (*P*<0.05; Fig. 6E). The interactive effects of elevated [CO_2_] and irrigation were highly significant in leaf area (*P*<0.05; Table 4) and aboveground biomass (*P*<0.05; Table 4).

### Yield and ear characteristics of maize

The study also revealed that when irrigation was compared with the natural precipitation at 390, 450 and 550 µmol·mol^−1^ [CO_2_] levels, significantly increased the seed yield by 17.48%, 14.91% and 10.59%, respectively (*P*<0.05; Table 3). There was also a significant increase in biological yield by 12.39%, 9.30% and 8.39%, respectively (*P*<0.05; Table 3). Following from the above, economic coefficient showed a significant increase that was higher in irrigation than natural precipitation at each [CO_2_] level (*P*<0.05; Table 3). There were significant interactive effects of elevated [CO_2_] and irrigation on maize seed yield (*P*<0.05; Table 4) and biological yield (*P*<0.01; Table 4).

**Table 3:** Multiple comparison on yield of maize under effects of elevated [CO_2_] and irrigation.

Treatments	Seed yield /(g/plant)	Increase compare with its CK %	Biology yield /(g/plant)	Increase compare with its CK %	Economical coefficient
C_550_W_+15%_	336.76±2.75^a^	10.59	617.34±5.23^a^	8.39	0.55±0.01^a^
C_550_W_0_(CK)	304.52±1.01^c^		569.55±1.19^c^		0.53±0.00^a^
C_450_W_+15%_	314.34±4.22^b^	14.91	585.81±3.84^b^	9.30	0.54±0.00^a^
C_450_W_0_(CK)	273.55±5.52^e^		535.98±2.10^e^		0.51±0.01^b^
C_390_W_+15%_	281.53±6.35^d^	17.48	554.18±3.78^d^	12.39	0.51±0.01^b^
C_390_W_0_(CK)	239.64±1.09^f^		493.09±5.54^f^		0.49±0.00^c^

The data are means ± SD (n = 3).

Different lower cases letters (a,b,c,d,e,f) indicated significant difference (*P*<0.05).

**Table 4:** The two-way analysis of variances on growth and yield of maize between different elevated [CO_2_] and irrigation treatment.

Variable	CO_2_ concentration			Irrigation			Interaction		
	*df*	*F*	*P*	*df*	*F*	*P*	*df*	*F*	*P*
Plant height	2	536.048	0.000	1	1105.743	0.000	2	6.507	0.012*
Ear height	2	9.315	0.002	1	26.167	0.000	2	0.726	0.498
Stem diameter	2	0.988	0.392	1	2.454	0.135	2	0.020	0.980
Leaf area	2	622.766	0.000	1	777.613	0.000	2	4.821	0.029*
Aboveground biomass	2	58.838	0.000	1	137.036	0.000	2	4.339	0.038*
Seed yield	2	663.180	0.000	1	806.085	0.000	2	5.113	0.017*
Biology yield	2	945.868	0.000	1	1627.130	0.000	2	9.953	0.001**

* and ** indicated *P*<0.05 and *P*<0.01, respectively.

The maize ear characteristics showed significant differences in six treatments under elevated [CO_2_] and irrigation (*P*<0.05; Table 5). Irrigation increased 100-kernel weight by 10.59%, 8.20% and 5.19% at 390, 450 and 550 µmol·mol^−1^ [CO_2_] respectively, whereas that of shriveled kernels decreased by 70.33%, 74.70% and 73.68%, respectively. Kernels per row and kernel number of C_390_W_+15%_, C_450_W_+15%_ and C_550_W_+15%_ were significantly increased more than C_390_W_0_, C_450_W_0_ and C_550_W_0_ (*P*<0.05). Also, rows per ear increased, but there was no difference among the six treatments. Maize ear length, ear diameter and ear weight were all significantly increased under irrigation, whereas axle diameter showed increasing trend at 390, 450 and 550 µmol·mol^−1^ [CO_2_] levels (*P*<0.05). However, there was no noteworthy difference for bare-tip length (*P*<0.05; Table 5).

**Table 5:** Change on ear characteristics of maize under effects of elevated [CO_2_] and irrigation.

Treatments	Ear length (cm)	Ear diameter (cm)	Ear weight (g)	100-kernel weight (g)	Kernels per row (kernels/row)	Kernel number (No.)	Rows per ear (row/ear)	Shriveled kernels (kernels/ear)	Bare-tip length (cm)	Axle diameter (cm)
C_550_W_+15%_	24.17±0.35^a^	6.16±0.04^a^	362.45±0.93^a^	43.97±0.21^a^	44.67±1.15^a^	820.67±2.31^a^	18.67±1.15^a^	5.00±1.00^e^	4.51±0.04^bc^	3.80±0.31^a^
C_550_W_0_(CK)	22.21±0.05^c^	5.97±0.04^b^	332.28±16.86^b^	41.80±0.40^b^	41.33±0.58^b^	757.67±1.53^b^	17.33±1.15^a^	19.00±1.00^c^	4.45±0.04^c^	3.72±0.37^ab^
C_450_W_+15%_	23.05±0.45^b^	6.03±0.04^b^	344.69±4.08^b^	42.60±0.50^b^	42.33±0.58^b^	762.00±1.39^b^	18.00±0.01^a^	7.00±1.00d^e^	4.42±0.04^c^	3.64±0.28^bc^
C_450_W_0_(CK)	21.02±0.12^d^	5.57±0.01^d^	305.56±4.07^c^	39.37±0.84^c^	38.33±0.58^c^	614.67±3.06^d^	17.33±1.15^a^	27.67±2.08^b^	4.62±0.01^b^	3.61±0.31^bc^
C_390_W_+15%_	21.25±0.03^d^	5.69±0.17^c^	312.01±1.77^c^	40.40±0.75^c^	39.00±1.00^c^	676.00±1.58^c^	17.33±1.15^a^	9.00±1.00^d^	4.44±0.17^c^	3.55±0.26^c^
C_390_W_0_(CK)	19.28±0.35^e^	5.17±0.04^e^	254.25±1.45^d^	36.53±1.31^d^	35.67±0.58^d^	594.33±2.89^e^	16.67±1.15^a^	30.33±1.15^a^	4.87±0.04^a^	3.54±0.28^c^

The data are means ± SD (n = 3).

Different lower cases letters (a,b,c,d,e) indicated significant difference (*P*<0.05).

## Discussion

### Interactive effects on photosynthetic parameters of maize

The response of plant photosynthesis to each of the environmental variables (e.g., water availability, temperature, nitrogen) associated with the elevated [CO_2_] has not been sufficiently understood [55]–[58]. The present study indicated that leaf *P*_n_ of maize improved with both elevated [CO_2_] and irrigation, and the curves of C_550_W_+15%_, C_550_W_0_, C_450_W_+15%_, C_450_W_0_ and C_390_W_+15%_ showed higher values than C_390_W_0_ curve (Fig 5A). Similar results have been found in studies of *Stipa breviflora*, which reported a significant increase in *P*_n_ under elevated [CO_2_] and increased precipitation of 15% [59], and wall *et al*. (2001) also observed that elevated [CO_2_] increased *P*_n_ of sorghum by 9% in wet condition in FACE [60]. Leakey (2006) indicated that elevated [CO_2_] can increase plant photosynthetic capacity and yield by adjusting its water state, so elevated [CO_2_] will have positive effect in water deficit condition [61]. In this experimental site, the relative soil moisture of natural precipitation is lower as compared with irrigation (Fig. 3) and in-fact under slight drought, and it implied that water deficit is a key factor limiting maize growth. In contrast to *P*_n_, *T*_r_ decreased with elevated [CO_2_], and much lower in natural precipitation treatment than irrigation treatment at each [CO_2_] level (Fig 5B). A number of other controlled environment studies [62]–[63] all observed the *T*_r_ decreased at elevated [CO_2_]. It is worth mentioning, that the decrease of *T*_r_ was associated with decrease of *G*_s_, when elevated [CO_2_] decreased leaf *G*_s_, and caused increasing resistance from intrinsic leaf to outside, resulting in the decrease of *T*_r_ [64]. Additionally, the studies of elevated [CO_2_] and drought on plant reported that elevated [CO_2_] declined *T*_r_ in low soil moisture than high soil moisture [25], [62], which in accordance with our findings. This suggested that elevated [CO_2_] may increased stomatal resistance and leaded to the reduce of *T*_r_ [25], but irrigation have a positive effect on stomatal opening and *T*_r_ have a increased trend in irrigation treatment compare with natural precipitation treatment at each [CO_2_] level. Moreover, our data indicated that *WUE* increased with elevated [CO_2_], and higher in natural precipitation treatment than irrigation treatment at elevated [CO_2_] levels (Fig 5C). This is in agreement with the results of previous studies, which reported elevated [CO_2_] have an increase trend in *WUE* under drought condition than well-watered condition [27], [61], [62], [65]. Specifically, we found that the *WUE* have a decrease trend in irrigation treatment than natural precipitation treatment at elevated [CO_2_] levels, whereas the yield of that increased. This is mainly due to the increase of leaf area and biomass of crop community under elevated [CO_2_] and irrigation, resulting in the increase of *WUE* of crop community, at last performance increase in yield of maize [25], [66]–[69]. In our study, *C*_i_ increased with elevated [CO_2_], whereas irrigation caused a small increase of *C*_i_ at each [CO_2_] level (Fig 5E). However, *G*_s_ decreased with elevated [CO_2_], and irrigation increased it greatly at each [CO_2_] level (Fig 5D). Some controlled environment studies [70]–[72] also showing that elevated [CO_2_] could cause a decrease in plant stomatal conductance (*G*_s_) and partly closing of the stomata. Curtis *et al.* (1998) found that doubling [CO_2_] average reduced *G*_s_ by 11% [73]. Further, the decreased *G*_s_ might be associated with the increase in *C*_i_, because a prior work showing that, *C*_i_ increased with rising [CO_2_], wheat adjusted stomata opening width will bring about a decrease in *C*_i_ so as to keep the intercellular CO_2_ partial pressure to be always lower than atmospheric CO_2_ partial pressure [74].

Photosynthetic parameter represented photosynthetic capacity and efficiency [75], which was usually obtained from irradiance-response model. Ye and Yu (2008) indicated that the modified rectangular hyperbolic model fitting results were quite close to the real values compare with the other models by series verification test, and the main photosynthetic parameters can be directly generated without any assumption by the model [52]–[53]. The model mentioned above was used and the *P*_n_ curves were fitted, and producing the main parameters with R^2^>0.99 (Table 2). Irrigation increased *P*_nmax_ by 12.43%, 19.8% and 29.70% at 390, 450 and 550 µmol·mol^−1^ [CO_2_] levels, respectively (Table 2). The rise in *P*_nmax_ has shown an improvement concerning the photosynthetic electron transport rate and photophosphorylation activity. It has also shown an improvement in the photosynthetic capacity of maize. In addition, *LSP* increased by 9.51%, 7.15% and 8.25% under irrigation more than natural precipitation at 390, 450 and 550 µmol·mol^−1^ [CO_2_] levels, respectively (Table 2). The emergence of *LSP* is actually as a result of dark reactions which are able to keep up with light reactions under the intense radiation, thus restricting the increase of photosynthetic rate. However, additional irrigation under elevated [CO_2_] might partly alleviate the negative effect and could promote photosynthetic capacity of maize under high light intensity. Normally, the photosynthesis reaction in a plant is very weak when in *LCP*, thus there was no significant effect on maize photosynthesis, even after there was a change in *LCP* with elevated [CO_2_] and irrigation (Table 2). In addition, irrigation increased *R*_d_ at each [CO_2_] level, which is the limiting factor for maize photosynthesis. Generally, elevated [CO_2_] can cause an increase in temperature, and make plant respiration quickened, leading to increase in consumption. Thus, the net effect is an increase in *R*_d_ [70]. Irrigation might enhance this effect, but further researches are needed to explore the reasons.

### Interactive effects on growth of maize

The plant height increased by 5.28%, 4.60% and 5.86% under conditions of irrigation as compared with natural precipitation at 390, 450 and 550 µmol·mol^−1^ [CO_2_] levels, respectively (Fig. 6A). Similar results could be found for ear height (Fig. 6B), whereas stem diameter did not show any significant difference (Fig. 6C). Significant interactive effects of elevated [CO_2_] and irrigation were observed in plant height, whereas there was no significant interactive effects of elevated [CO_2_] and irrigation in ear height and stem diameter of maize (*P*<0.05; Table 4). Some studies reported that leaf area of maize significantly increased under elevated [CO_2_] [76]–[77], and this research work is in agreement with those findings (Fig. 6D). In addition, irrigation increased leaf area by 12.04%, 9.90% and 7.75% at 390, 450 and 550µmol··mol^−1^ [CO_2_] levels, respectively (Fig. 6D). A similar conclusion could be found in *Stipa breviflora*, in which, the leaf area increased to a maximum under conditions of elevated [CO_2_] and increased precipitation of 15% [78]. Chamber studies showed that changing [CO_2_] has no effect on biomass of maize and sorghum under adequate moisture conditions [79]–[81], and even the negative effects [82]. However, this study found that aboveground biomass of maize significantly increased by 16.66%, 10.75% and 7.65% under irrigation more than natural precipitation at 390, 450 and 550 µmol·mol^−1^ [CO_2_] levels, respectively (Fig. 6E). This result is consistent with Cure's findings [83], who reported that maize aboveground biomass increased by 7% under well-watered more than dry conditions at 550µmol··mol^−1^ [CO_2_] in chamber (n = 4). Shi *et al.* (2013) observed that the aboveground biomass of *Stipa breviflora* significantly increased with elevated [CO_2_] and increasing precipitation [78]. Moreover, There were significant interactive effects of elevated [CO_2_] and irrigation in leaf area (*P*<0.05; Table 4) and aboveground biomass (*P*<0.05; Table 4).

### Interactive effects on yield and ear characteristics of maize

The results of that elevated [CO_2_] increased maize yield have been indicated in previous studies. Cure *et al.* (1986) reported that maize average yield increased by 27% (n = 3) by doubling [CO_2_] [83], while Guo (2003) indicated that maize yield might be increased by 22.88% when [CO_2_] levels rise up to 700µmol··mol^−1^ [84]. Long *et al.* (2006) used comprehensive observation data of chamber studies (n = 14) and found out that grain yield of maize and sorghum increased by an average of 18% when [CO_2_] were elevated to 550 µmol·mol^−1^ [85]. This study has exhaustively shown that the maize yield of Northeast China increased by 14.15% and 27.07% with 450 and 550 µmol·mol^−1^ [CO_2_] levels, respectively (Table 3). Additionally, irrigation significantly increased maize yield by 10.59%, 14.91% and 17.48% as compared with natural precipitation at 390, 450 and 550 µmol·mol^−1^ [CO_2_] levels, respectively (Table 3). Similar results have been reported in previous studies [86]–[87], but others have reported decreases [82] or no significant change at all [79]–[80]. Allen (2011) predicted that management of irrigation water in a future high [CO_2_] world could potentially increase overall C_4_ crop yield (in water-limited areas) [62], we agree with this idea. Moreover, biological yield has also been increased by 8.39%, 9.30% and 12.39% under irrigation more than natural precipitation at 390, 450 and 550 µmol·mol^−1^ [CO_2_] levels, respectively (Table 3). The work also revealed that there has been significant interactive effects of elevated [CO_2_] and irrigation on maize seed yield (*P*<0.05; Table 4) and biological yield (*P*<0.01; Table 4). Consequently, maize economic coefficient increased in irrigation more than natural precipitation. Economic coefficient reflects the transport and store ability of crop “source” to “sink” form photosynthetic products [88]. Kirschbaum (2010) reported that plant growth response to elevated [CO_2_] increase with a plant's sink capacity and nutrient status [89]. In present study, maize photosynthetic capacity was enhanced while the ear capacity was expanded under elevated [CO_2_] and irrigation, resulting in a more dry matter accumulation and yield increase.

Maize ear characteristics significantly changed under the interactive effects of elevated [CO_2_] and irrigation. 100-kernel weight and kernal number were all significantly increased under irrigation than natural precipitation at each [CO_2_] level (Table 5). Additionally, ear length, ear diameter and ear weight were all increased in accordance with yield, and the increase of 100-kernel weight and ear length was consistent with the study done by Wang et al. (1996) [90]. There is a study reported that kernels per row is the main factor that will increase maize yield [91]. In this study, irrigation increased kernels per row by 9.34%, 10.44% and 8.08% at 390, 450 and 550 µmol·mol^−1^ [CO_2_] levels respectively, whereas rows per ear change was not remarkable (Table 5). The decreased of shriveled kernels was beneficial to yield increase. In our study, irrigation decreased shriveled kernels by 70.33%, 74.70% and 73.68% at 390, 450 and 550 µmol·mol^−1^ [CO_2_] levels, respectively (Table 5). Optimization of the ear characteristics were reflected in an increase in yield under elevated [CO_2_] and irrigation. Therefore, it is necessary to irrigate with additional water for the elevated [CO_2_] plots of maize to compensate for photosynthesis resistance from lacking of water under elevated [CO_2_] condition. Moreover, bare-tip length of maize made no significant difference in all treatments, but axle diameter increased within a narrow range. The increase was by 0.28%, 0.83% and 2.15% in irrigation more than natural precipitation at 390, 450 and 550 µmol·mol^−1^ [CO_2_] levels (Table 5). This change implied that elevated [CO_2_] and irrigation increased ear length, ear diameter as well as axle diameter. Thus, it can be estimated that maize yield could not be increased unlimitedly with elevated [CO_2_] and irrigation, but increasing the axle diameter serves as a limiting factor.

## Conclusions

Our results demonstrated the following: 1) With elevated [CO_2_] and irrigation, leaf *P*_n_ and *C*_i_ of maize significantly increased, while elevated [CO_2_] brought a notable decrease in *G*_s_ and *T*_r_, but irrigation have a positive effect on them, thereby increasing *WUE* in natural precipitation treatment than irrigation treatment at elevated [CO_2_] levels. 2) Irradiance-response parameters *P*_nmax_ and *LSP* increased more under irrigation conditions than natural precipitation at each [CO_2_] level. *R*_d_ also increased under the same conditions, and this is a limiting factor. 3) Irrigation under elevated CO_2_ increased plant height, ear height, stem diameter, leaf area and aboveground biomass, resulting in the increase of yield. In addition, ear characteristics of maize were all superior except axle diameter. However, further study should be taken to ensure the contribution rate of elevated [CO_2_] and irrigation.
