# Correction: Interactive Effects of Elevated CO_2_ Concentration and Irrigation on Photosynthetic Parameters and Yield of Maize in Northeast China

**DOI:** 10.1371/journal.pone.0105898

**Published:** 2014-08-14

**Authors:** The PLOS ONE Staff

The affiliations of the corresponding author Jiahua Zhang are incorrect. The correct affiliations are: (3) Key Laboratory of Digital Earth Science, Institute of Remote Sensing and Digital Earth, Chinese Academy of Sciences, Beijing, China and (1) Institute of Eco-Environment and Agro-Meteorology, Chinese Academy of Meteorological Sciences, Beijing, China.
